# Mindfulness-based therapy for insomnia alleviates insomnia, depression, and cognitive arousal in treatment-resistant insomnia: A single-arm telemedicine trial

**DOI:** 10.3389/frsle.2023.1072752

**Published:** 2023-03-08

**Authors:** David A. Kalmbach, Philip Cheng, Jason C. Ong, Anthony N. Reffi, David M. Fresco, Cynthia Fellman-Couture, Melissa K. Ruprich, Zain Sultan, Chaewon Sagong, Christopher L. Drake

**Affiliations:** ^1^Thomas Roth Sleep Disorders and Research Center, Henry Ford Health, Detroit, MI, United States; ^2^Center for Circadian and Sleep Medicine, Department of Neurology, Northwestern University Feinberg School of Medicine, Chicago, IL, United States; ^3^Behavioral Sleep Medicine, Nox Health, Suwanee, GA, United States; ^4^Department of Psychiatry, University of Michigan, Ann Arbor, MI, United States

**Keywords:** sleep, MBTI, perseverative thinking, rumination, CBT-I, clinical trial, meditation

## Abstract

**Objectives:**

Cognitive-behavioral therapy and pharmacotherapy are effective insomnia treatments, yet half of patients do not remit. Emerging evidence indicates refractory cognitive arousal is associated with poor insomnia treatment outcomes, giving rise to the concept that therapeutic approaches directly aimed at reducing cognitive arousal may benefit patients with a history of inadequate response to intervention. This proof-of-concept study examined the effects of mindfulness-based therapy for insomnia (MBTI) delivered individually *via* telemedicine on insomnia, depression, and cognitive arousal in patients with treatment-resistant insomnia.

**Methods:**

A single-arm trial wherein 19 patients whose insomnia did not remit with prior psychotherapy and/or pharmacotherapy received a course of MBTI as second-stage therapy, which included eight weekly 1-h sessions in an individual format *via* telemedicine video. Study outcomes included the 15-item version of the five-facet mindfulness questionnaire (FFMQ-15), insomnia severity index (ISI), Patient Health Questionnaire-9 to assess depression (PHQ-9), and three cognitive arousal indices: pre-sleep arousal scale's cognitive factor, perseverative thinking questionnaire, and the daytime insomnia symptom response scale.

**Results:**

Patients reported increased mindfulness from pretreatment to posttreatment (FFMQ-15: 52.95 ± 8.30 to 57.47 ± 9.82, *p* = 0.008). Patients also reported large reductions in ISI (16.42 ± 3.95 to 8.37 ± 4.19, *p* < 0.001, Cohen's dz = 1.73; 57.9% remission), PHQ-9 (6.42 ± 3.47 to 3.32 ± 2.93, *p* = 0.001, Cohen's dz = 0.93), and all cognitive arousal indices (Cohen's dzs = 0.82–1.30) at posttreatment. Six months later, ISI scores and cognitive arousal levels remained significantly lower than pretreatment, although effect sizes decreased for ISI (Cohen's dz = 1.11) and cognitive arousal (Cohen's dzs = 0.63–0.68). Antidepressant effects were no longer significant at follow-up.

**Conclusion:**

Treatment-resistant insomnia patients are engaged in MBTI, which produces large acute reductions in insomnia, depression, and cognitive arousal. MBTI effects on insomnia and cognitive arousal were moderate to large 6 months after treatment. These findings support the concept and feasibility of MBTI for treatment-resistant patients along with indication that longer-term strategies are needed to help maintain acute treatment gains.

**Clinical trial registration:**

ClinicalTrials.gov, identifier NCT03724305.

## Introduction

Cognitive-behavioral therapy for insomnia (CBTI) is the guideline recommended treatment for insomnia disorder (Qaseem et al., 2016), which produces remission rates of 40–65% in a wide range of patient populations (Morin et al., 2009; Harvey et al., 2014; Wu et al., 2015; Drake et al., 2019; Manber et al., 2019; Arnedt et al., 2021). Pharmacotherapy is the most widely delivered insomnia intervention in real-world practice but is less effective than CBTI as reflected by remission rates of <50% (Gross et al., 2011; Pillai et al., 2017; Morin et al., 2020). Patients treated with a combination of psychotherapy and pharmacotherapy achieve insomnia remission rates of 44–62% (Manber et al., 2008; Morin et al., 2009). Taken together, about half of patients who receive empirically supported insomnia intervention—whether cognitive, behavioral, pharmacological, or a combination—continue having unresolved clinical symptoms after treatment, which are associated with higher rates of depression, suicidal thoughts, and lower quality of life (Trockel et al., 2015; Kalmbach et al., 2019a,b, 2022a,c). Unfortunately, little is known about efficacious treatment options for insomnia non-remitters, thereby leaving no clear path to recovery for patients who do not sufficiently respond to initial intervention.

Recent evidence supports sequencing interventions for insomnia patients who do not adequately respond to initial treatment efforts. Morin and colleagues showed that patients whose insomnia did not remit with initial behavior therapy subsequently benefited from second-stage therapy in the form of zolpidem (remission increased from 38% to 56%) or cognitive therapy (remission increased from 38% to 45%) (Morin et al., 2020). Similarly, patients whose insomnia did not remit with zolpidem had small (even if non-significant) increases in remission after second-stage therapy with trazodone (31%−49%) or behavior therapy (29%−36%). These data support sequencing insomnia therapies to enhance patient outcomes, yet remission rates even after sequencing were still about 50% across groups. To optimize patient care, unmet clinical needs after first-stage therapy should guide insomnia patients to the appropriate second-stage therapy, which may improve overall remission rates.

### Poor insomnia treatment response is linked to refractory cognitive arousal

Myriad clinical trials have attempted to identify pretreatment characteristics—e.g., sociodemographics, clinical presentations, etc.—that moderate patient outcomes in insomnia treatment. Unfortunately, sociodemographics do not reliably predict insomnia treatment response, and moderating results for comorbid conditions and objective sleep disturbances on insomnia treatment outcomes are mixed (Espie et al., 2001; Troxel et al., 2013; Inoue et al., 2015; van de Laar et al., 2015; Miller et al., 2018; Cheng et al., 2019; Rochefort et al., 2019; Kalmbach et al., 2020b; Pruiksma et al., 2020; Galbiati et al., 2021; Edinger et al., 2022).

However, recent evidence suggests that alleviations in cognitive arousal enhance insomnia treatment outcomes. High cognitive arousal—i.e., the transdiagnostic phenomenon of heightened cognitive activity, particularly in the form of perseverative thinking—is implicated in insomnia etiology, maintenance, and severity (Harvey, 2002; Fernandez-Mendoza et al., 2010). Prior clinical trials show that reducing cognitive arousal enhances CBTI effects on insomnia and comorbid depression (Vincent and Walsh, 2013; Espie et al., 2014; Sunnhed and Jansson-Fröjmark, 2015; Cheng et al., 2020). Despite the importance of this therapeutic mechanism, evidence suggests that standard CBTI yields mixed results for cognitive arousal and likely produces modest effects on these symptoms (Vincent and Walsh, 2013; Espie et al., 2014; Kalmbach et al., 2019a, 2020a, 2022c; Cheng et al., 2020).

As nearly all insomnia patients present with high cognitive arousal before treatment, our data show that pretreatment cognitive arousal does not differentiate between treatment responders and non-responders. Rather, *refractory* cognitive arousal—i.e., high cognitive arousal that does not lessen with treatment—is associated with poor insomnia treatment response (Ong et al., 2009; Kalmbach et al., 2019a, 2022c). In a sample of 658 US adults with DSM-5 insomnia disorder, a comparison of CBTI patients who reported reductions in cognitive arousal vs. those who did not (unpublished results from a RCT; Cheng et al., 2019) showed that patients with refractory cognitive arousal (no reduction in arousal) were two times less likely to remit than those whose arousal decreased with CBTI (31% vs. 65%). In a sample of pregnant women with insomnia, we found that patients with high nocturnal cognitive arousal after CBTI were four times less likely to remit from insomnia than patients whose arousal decreased with CBTI; unreported secondary analysis data from our prior randomized controlled trial (RCT) (Kalmbach et al., 2020a, 2022c).

Refractory cognitive arousal may not only be a barrier in insomnia treatment. Empirical data show that individuals vary in the degree to which their cognitive arousal levels change over time (Bean et al., 2020, 2021). In other words, some people have flexible cognitive arousal levels that may reflect attempts to regulate stress (e.g., Person A has low cognitive arousal, but then they experience a stressor which increases perseveration on that stressor), whereas others have cognitive arousal that resists change over time (e.g., Person B has persistently high cognitive arousal even in the absence of external stressors). Daily diary data show that individuals whose cognitive arousals levels are resistant to change over time (i.e., refractory cognitive arousal) experience higher levels of stress-related pathology—namely, depression—in prospective observational research (Bean et al., 2020). Specifically, individuals with refractory cognitive arousal are at increased risk for mental illness due to a theorized inability to adapt to stress (Bean et al., 2020, 2021).

Taken together, high cognitive arousal is associated with insomnia, and some patients' cognitive arousal levels are more resistant to change. As a result, some patients report decreased cognitive arousal with first-line insomnia treatment, and these patients experience favorable outcomes. However, many patients do not experience clinically meaningful reductions in cognitive arousal with treatment, and these patients are unlikely to maximally benefit from insomnia treatment. These data suggest that initial non-remitters may benefit from a second-stage therapy that reliably produces large reductions in cognitive arousal.

### Mindfulness-based therapy for insomnia

Cognitive arousal can be difficult to treat, and high levels of rumination and worry are linked to poor prognosis in therapy (Watkins, 2008). However, mindfulness-based interventions targeting metacognitive processes (thinking about thinking) and decentering (ability to observe thoughts and feelings as temporary) yield success in this area (see Ong et al., 2012 for review). Higher levels of mindfulness correspond to lower levels of cognitive arousal at night (Ong et al., 2012; Kalmbach et al., 2020c), and mindfulness-based interventions are among the most effective methods to reduce perseverative thinking (Deyo et al., 2009; Campbell et al., 2012; Querstret and Cropley, 2013; Creswell, 2017; Renna et al., 2017, 2018; Winnebeck et al., 2017). Indeed, mindfulness-based interventions enhance patient outcomes in treatment-resistant depression by reducing cognitive arousal (typically in the form of rumination) and by increasing decentering (Kenny and Williams, 2007; Eisendrath et al., 2008, 2011; Moore et al., 2022).

Mindfulness-based therapy for insomnia (MBTI) was developed as an alternative to CBTI by using mindfulness practices to reduce cognitive arousal, and integrating behavioral sleep strategies (e.g., sleep consolidation, stimulus control) within a mindfulness-based intervention framework (Ong, 2017). In a single-arm trial of MBTI, patients reported large reductions in nocturnal cognitive arousal (Cohen's d = 0.95) and insomnia (Cohen's d = 1.32) (Ong et al., 2008). A follow-up RCT compared efficacy of MBTI and mindfulness-based stress reduction (MBSR) to a control condition for chronic insomnia (Ong et al., 2014). MBTI and MBSR produced similar reductions in nocturnal arousal relative to control, whereas MBTI produced larger longer-term benefits for sleep symptoms. These reliably large effects of MBTI on cognitive arousal contrast with other RCTs (digital and face-to-face) showing that standard CBTI produces more modest or even sometimes non-significant therapeutic benefit for cognitive arousal symptoms (Vincent and Walsh, 2013; Espie et al., 2014; Cheng et al., 2019; Kalmbach et al., 2019a, 2020a, 2022c). Given the emerging findings on refractory cognitive arousal as a link to poor treatment response, MBTI may serve as a viable second-stage therapy for patients who do not adequately respond to first-stage therapy with CBTI and/or pharmacotherapy.

The present study was a single-arm proof-of-concept clinical trial testing the effects of MBTI on insomnia, depression, and cognitive arousal in patients with treatment-resistant insomnia. In the present study, we operationalized treatment resistance as clinically significant insomnia symptoms after inadequate response to at least one trial of psychotherapeutic and/or pharmacotherapeutic intervention for insomnia. In accordance with clinic operations and patient expectations since the COVID-19 pandemic began, MBTI was delivered *via* telemedicine video with a therapist. We examined acute treatment effects on insomnia, depression, and cognitive arousal from pretreatment to posttreatment, as well as longer term effects at 6-month follow-up. We hypothesized that treatment-resistant patients receiving telemedicine MBTI would report significant acute reductions in insomnia symptoms, depressive symptoms, and cognitive arousal. We also hypothesized that symptom levels would remain significantly lower than pretreatment levels 6 months after completing treatment.

## Materials and methods

### Ethical consideration

Ethical approval was obtained from the Institutional Review Board of Henry Ford Health. Informed written consent was obtained from patients before participation. All patients were informed of the voluntary nature of the study and assured of anonymity and confidentiality.

### Participants and recruitment sources

Patients were recruited from two sources. Our first source was a pool of patients who received stepped-care treatment wherein digital CBTI was administered to all insomnia patients in this program, and patients who did not remit with digital CBTI were then offered clinician-led CBTI. Specifically, we invited 40 patients who did not remit in stepped-care CBTI, and 19 of these patients expressed interest in participating. After eligibility screening (see criteria below), 17 of these patients were enrolled into our MBTI trial.

Our second source of recruitment was physician referrals within our health system. Specifically, physicians with awareness of our insomnia clinical trials research reached out to our team with a total of two patients whose insomnia was poorly controlled with pharmacotherapy. Both patients were screened for eligibility and were enrolled based on insomnia severity, and patient and physician reports of insufficient treatment response to pharmacotherapy. Thus, a total of *n* = 19 treatment-resistant patients participated in this single-arm open label trial.

#### Eligibility screening

Patients interested in our proof-of-concept clinical trial contacted the study team for a phone call to discuss study details with a nurse. Those who wished to participate after learning study details were directed to complete an online screening survey to determine study eligibility. A total of 21 patients were screened across two recruitment sources. Inclusion criteria were age ≥ 18, clinically significant insomnia symptoms (Insomnia Severity Index ≥ 11), and inadequate response to prior insomnia intervention per patient report (i.e., patients felt their insomnia did not resolve with prior treatment, which would be a real-world motivator for continued treatment). Exclusion criterion was currently ongoing cognitive and/or behavioral intervention for insomnia (current sleep aid use was not exclusionary). Of the 21 screened patients, one patient was ineligible due to not meeting inclusion for insomnia symptom severity, whereas another patient was deemed eligible, but did not enroll into MBTI due to scheduling conflicts.

### Procedures and study outcomes assessment schedule

**Figure 1:**
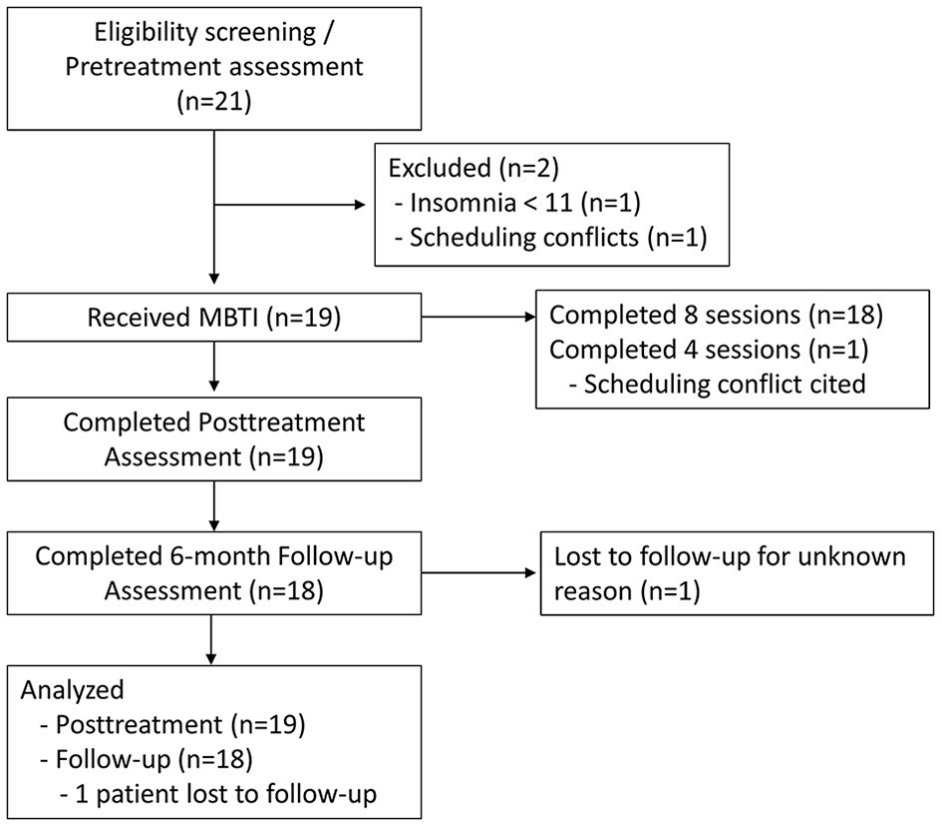
CONSORT flow diagram.

See Figure 1 for CONSORT flow diagram. Screening surveys served as pretreatment assessment for study outcomes, treatment history, sociodemographics, and other health information. Within a week of enrolling in the trial, patients underwent a clinical intake interview *via* telemedicine video with the nurse therapist to further assess insomnia presentation and family and social history. A week after the clinical interview, patients began MBTI treatment, which includes eight weekly sessions (described below). Posttreatment surveys were administered a week after the final MBTI session. Follow-up surveys were administered 6 months after posttreatment surveys were completed.

### Study intervention

*Mindfulness-based therapy for insomnia (MBTI)* is an evidence-based practice that places behavioral sleep strategies within a mindfulness-based intervention framework. MBTI was delivered in accordance with the clinician manual (Ong, 2017), but modified for individual therapy format. MBTI is traditionally administered in eight weekly 2-hr group sessions. However, treatment format was modified for this study to be delivered across eight weekly 1-hour sessions in an individual one-on-one format with a therapist; reduction in session time was afforded by reducing the length of discussing meditations (e.g., no group discussion) and by reducing the length of in-session meditations. The nurse therapist providing MBTI had been previously trained in behavioral sleep medicine (i.e., CBTI) and mindfulness-based stress reduction (MBSR) before training in MBTI. For the present study, the nurse was supervised by a licensed clinical psychologist with expertise in behavioral sleep medicine and mindfulness-based interventions for insomnia. Behavioral components of MBTI include sleep consolidation and stimulus control, whereas mindfulness elements include guided mindfulness meditations and engaging in mindful activities. To practice guided meditations at home, patients downloaded the Insight Timer app. After each session, the nurse therapist would email session summaries to patients, which included a specific meditation within the app to practice at home throughout the week; patients were also invited to explore other meditations available through the app. For full session-by-session details, please refer to the MBTI manual (Ong, 2017).

### Blinding

Participants were not blind to their intervention and were aware of the nature of MBTI. However, patients were blind to specific hypotheses, nor were they informed of our interest in the role of cognitive arousal in treatment-resistant insomnia.

### Measures

All study outcomes were assessed *via* online surveys hosted by Qualtrics.

*Sociodemographic information and history of insomnia treatment* were assessed during screening. Patients reported age, gender, race, annual household income, prior insomnia therapy and pharmacotherapy, and current medications including prescription (Rx) and over-the-counter (OTC) sleep aids. Medications were also assessed at posttreatment and follow-up assessment.

*Treatment engagement* was assessed *via* session attendance with eight sessions being the maximum number of sessions.

*Treatment adherence* regarding the guided meditation practices was assessed at posttreatment *via* patient reports of days per week they meditated at home during treatment and estimated minutes per day they meditated. For descriptive purposes, we assessed the time of day that patients meditated. Regarding adherence to behavioral sleep strategies, patients reported their perceptions of their ability to adhere to prescribed sleep schedules on a Likert-type scale with 1 = Not at all, 2 = Slightly, 3 = Moderately, 4 = Mostly, and 5 = Completely.

*The 15-item version of the Five Facet Mindfulness Questionnaire (FFMQ-15)* (Baer et al., 2012) was used to measure general trait-like tendency to be mindful in daily life. The FFMQ-15 was administered to test whether MBTI increased mindfulness in the sample. Scores on the FFMQ-15 range from 15 to 75 with higher scores indicating greater everyday mindfulness. The FFMQ-15 includes five scales including observing, describing, acting with awareness, non-judging, and non-reacting. Scores on each scale range from 3 to 15. Internal consistency for the FFMQ-15 total score was excellent (Cronbach's α = 0.82). Subscale internal consistencies ranged from good to outstanding for describing (Cronbach's α = 0.91), acting with awareness (Cronbach's α = 0.88), non-judging (Cronbach's α = 0.76), and non-reacting (Cronbach's α = 0.70), whereas the observing scale yielded poor internal consistency (Cronbach's α = 0.36).

*Insomnia Severity Index (ISI)* measured global insomnia symptom severity (Bastien et al., 2001; Morin et al., 2011), which was the primary end-point for sleep in this trial. Scores range from 0 to 28 with higher scores reflecting greater insomnia severity. ISI ≥ 11 indicates clinically significant insomnia symptoms in patient populations, which was required for study inclusion. Per standard practice, treatment remission was defined as ISI ≤ 7. The ISI had good internal consistency in the present study (Cronbach's α = 0.70).

*Sleep parameters* served as secondary end-points for sleep in this trial, which included patient reports of sleep latency, wake after sleep onset, and total sleep time. Sleep latency was assessed in response to: “On average, how long has it taken you to fall asleep within the last 2 weeks?” Wake after sleep onset was assessed in response to: “On average, how long did your awakenings in the middle of the night last during the past two weeks?” Total sleep time was assessed in response to: “Thinking about the past 2 weeks, how long did you actually sleep each night (not including time awake in bed)?”

*The Patient Health Questionnaire-9 (PHQ-9)* was used to assess depression (Spitzer et al., 1999). PHQ-9 scores range from 0-27 with higher scores indicating greater depression. PHQ-9 scores in the 1–4 range indicate minimal depression, whereas scores 5–9 indicate mild depression. The PHQ-9 ≥ 10 cutoff yields good sensitivity and specificity for detecting major depressive disorder. The PHQ-9 had good internal consistency in the present study (Cronbach's α = 0.73).

*The Pre-Sleep Arousal Scale's Cognitive factor (PSASC)* is a transdiagnostic measure that was used to assess nocturnal cognitive arousal (Nicassio et al., 1985), which served as the primary end-point for cognitive arousal. PSASC scores range from 8 to 40 with higher scores indicating greater cognitive arousal while trying to fall asleep (e.g., “can't shut off your thoughts”). PSASC ≥ 19 is a validated cutoff for detecting high nocturnal cognitive arousal (Puzino et al., 2019; Kalmbach et al., 2022b). The PSASC had excellent internal consistency in the present study (Cronbach's α = 0.84).

*The Perseverative Thinking Questionnaire (PTQ)* is a transdiagnostic measure that was used to assess general perseverative thinking (Ehring et al., 2011), which served as a secondary end-point for cognitive arousal. PTQ scores range from 0 to 60 with higher scores indicating greater perseverative thinking (e.g., “The same thoughts keep going through my mind again and again”). The PTQ had outstanding internal consistency in the present study (Cronbach's α = 0.96).

*The Daytime Insomnia Symptom Response Scale (DISRS)* was used to assess insomnia-focused rumination (Carney et al., 2013), which served as another secondary end-point for cognitive arousal. Unlike the PSASC and PTQ, the DISRS is not transdiagnostic, but rather specifically measures rumination on insomnia symptoms (e.g., “Think about how tired you feel”). Scores range from 20 to 80 with higher scores indicating greater insomnia-focused rumination. The DISRS had outstanding internal consistency in the present study (Cronbach's α = 0.93).

### Analysis plan

Study outcomes were downloaded directly from Qualtrics and all analyses were performed in SPSS version 26 (IBM Corp) with a significance value of 0.05. We first examined descriptive data for sample characteristics, including sociodemographics, insomnia treatment history, and presenting clinical symptoms. Study hypotheses were tested using a complete case analysis approach, which included all patients enrolled in MBTI. As the study was a single-arm proof-of-concept clinical trial, we conducted paired samples *t*-tests to test acute pretreatment to posttreatment effects of MBTI on mindfulness, sleep symptoms, depression, and cognitive arousal. To test long-term effects, we conducted paired samples *t*-tests comparing study outcomes at pretreatment and 6-month follow-up. Paired samples *t*-test effect sizes are expressed using Cohen's dz; small = 0.20, medium = 0.50, large = 0.80, very large ≥ 1.00. In addition, we conducted *post-hoc* analyses to further explore our data, which included examining frequency of Rx and OTC sleep aid use at posttreatment and follow-up (note: reducing sleep aid use can be a goal of insomnia therapy, but this was not the case in the present study; rather we observed spontaneous changes in sleep aid use), as well as rates of insomnia remission and major depression among patients with high vs. low cognitive arousal at posttreatment and follow-up.

## Results

### Patient characteristics and missing data

Full sample characteristics are reported in Table 1. Our sample consisted of adults aged 27–73 years and was predominantly female (78.9%). We observed good representation among those who identify as non-Hispanic White and non-Hispanic Black, but no other racial groups were represented. Patients were highly educated with over 2/3 having a 4-year college degree or higher level of education. Patients reported a median insomnia duration of 10 years.

**Table 1:** Sociodemographic information and insomnia treatment history (*n* = 19).

**Age (M ± SD; range)**	**57.16 yrs ± 14.69; 27–73**	**Years w/insomnia (M ± SD)**	**13.42 years ± 11.87**
Age (Median)	64 years	<1 year	1/19; 5.3%
Race		1–5 years	5/19; 26.3%
Non-Hispanic White (*n*; %)	12/19; 63.2%	6–10 years	4/19; 21.1%
Non-Hispanic Black (*n*; %)	7/19; 36.8%	11–15 years	2/19; 10.5%
Female gender (*n*, %)	15/19; 78.9%	16–20 years	5/19; 26.3%
Education (*n*, %)		>20 years	2/19; 10.5%
High school	4/19; 21.1%	Insomnia treatment history	
2-yr college or vocational school	2/19; 10.5%	Rx sleep aids only	2/19; 10.5%
Bachelor's degree	2/19; 10.5%	Digital CBTI	17/19; 89.5%
Some graduate school	4/19; 21.1%	Clinician-led CBTI	16/19; 84.2%
Master's degree	5/19; 26.3%	Current Rx sleep aid use	
Doctorate or professional deg	2/19; 10.5%	No	15/19; 78.9%
Annual household income		Yes	4/19; 21.1%
$20,000–24,999	2/19; 10.5%	Rx sleep aid	
$25,000–34,999	1/19; 5.3%	Zolpidem	2/4; 50.0%
$35,000–49,999	3/19; 15.8%	Temazepam	1/4; 25.0%
$50,000–74,999	3/19; 15.8%	Eszopiclone	1/4; 25.0%
≥$75,000	9/19; 47.4%	Rx sleep aid use duration	
Unreported	1/19; 5.3%	5–6 years	3/4; 75.0%
BMI (M ± SD; range)	26.37 ± 5.67; 19.4–39.5	>6 years	1/4; 25.0%
BMI ≥ 30 (*n*; %)	5/19; 26.3%	Current OTC sleep aid use	
		0	12/19; 63.2%
STOP-BANG (M ± SD; range)	2.37 ± 1.42; 0–6	1	2/19; 10.5%
STOP-BANG ≥ 3 (*n*; %)	9/19; 47.4%	2	2/19; 10.5%
		3	1/19; 5.3%
		4	1/19; 5.3%
		5 or more	1/19; 5.3%

M, mean; SD, standard deviation; n, number of participants; BMI, body mass index (kgm^2^); BMI ≥ 30 indicates obesity. STOP-BANG measures risk for obstructive sleep apnea; scores of 3 or higher indicate high risk. Rx, prescription; CBTI, cognitive behavioral therapy for insomnia; OTC, over-the-counter.

Regarding prior insomnia treatment, 16 patients previously completed two courses of CBTI (digital CBTI followed by clinician-led CBTI) and one patient completed a course of digital CBTI only, whereas two patients were referred by their sleep medicine physicians to our study after failing pharmacotherapy (i.e., no CBTI; Table 1). Over 1/3 of patients reported current OTC sleep aid use before treatment (*n* = 7/19; 36.8%), and 21.1% (*n* = 4/19) reported current Rx sleep aid use before treatment. All Rx sleep aid users reported a duration of use of 5 years or longer.

All patients reported study outcomes at posttreatment, but one participant did not provide 6-month follow-up data. The patient who did not provide follow-up data had previously reported insomnia remission, low cognitive arousal, and no depressive symptoms at posttreatment; he was not included in follow-up data analysis.

### Treatment engagement and adherence

See Table 2 for MBTI engagement and adherence metrics. Eighteen patients attended all eight MBTI sessions, whereas one patient discontinued MBTI after 4 sessions, citing scheduling conflicts. Patients reported high adherence to the daily at-home guided meditations with 78.9% (*n* = 15/19) practicing 6 or 7 days per week. Moreover, patients estimated meditating a mean of 39 mins per day with 31.6% meditating an hour or longer per day. Descriptive data suggest that meditating at home occurred similarly across morning, afternoon, evening, and at night before bed, whereas meditating in the middle of the night was uncommon. Patients reported high adherence to their prescribed sleep-wake schedule such that 73.7% (*n* = 14/19) indicating that they either “mostly” or “completely” adhered to their sleep schedules.

**Table 2:** Engagement and adherence metrics during MBTI.

**Number of sessions attended**	
4 sessions (*n*, %)	1/19; 5.3%
8 sessions (*n*, %)	18/19; 94.7%
At-home guided meditations	
Days per week (M ± SD)	6.05 ± 1.51
2 days per week (*n*, %)	1/19; 5.3%
3 days per week (*n*, %)	1/19; 5.3%
4 days per week (*n*, %)	1/19; 5.3%
5 days per week (*n*, %)	1/19; 5.3%
6 days per week (*n*, %)	4/19; 21.1%
7 days per week (*n*, %)	11/19; 57.9%
Minutes per day (M ± SD)	38.95 ± 18.53
15 mins per day (*n*, %)	2/19; 10.5%
20 mins per day (*n*, %)	3/19; 15.8%
25 mins per day (*n*, %)	1/19; 5.3%
30 mins per day (*n*, %)	4/19; 21.1%
40 mins per day (*n*, %)	1/19; 5.3%
45 mins per day (*n*, %)	2/19; 10.5%
≥60 mins per day (*n*, %)	6/19; 31.6%
Time of day for meditating (*n*, %)	
Morning	10/19; 52.6%
Afternoon	12/19; 63.2%
Evening	9/19; 47.4%
Night, before bed	10/19; 52.6%
Middle of night	1/19; 5.3%
Adherence to sleep schedule (*n*, %)	
Not at all	0/19; 0.0%
Slightly	1/19; 5.3%
Moderately	4/19; 21.1%
Mostly	11/19; 57.9%
Completely	3.19; 15.8%

M, mean; SD, standard deviation; n, number of participants.

### MBTI effects on mindfulness

Before examining MBTI effects on clinical symptoms, we tested whether MBTI increased mindfulness as intended (Table 3). A paired samples *t*-test revealed a medium-to-large increase in FFMQ-15 total scores (Cohen's dz = 0.71). When analyzing the individual FFMQ-15 subscales, we observed a nearly large increase in observing (Cohen's dz = 0.77) and a moderate-to-large increase in non-judging (Cohen's dz = 0.63). At 6-month follow-up, FFMQ-15 observing scores remained moderately higher than pretreatment (Cohen's dz = 0.52), whereas no other FFMQ-15 subscale at follow-up significantly differed from pretreatment.

**Table 3:** MBTI-related changes in mindfulness, sleep, depression, and cognitive arousal as tested by paired samples *t*-tests.

	**Pretreatment**	**Posttreatment**	***t*-statistic, *p*, Cohen's dz**	**6-m Follow-up**	**t-statistic, *p*, Cohen's dz**
**FFMQ-15**					
Total score (M ± SD)	52.95 ± 8.30	57.47 ± 9.82	***t*_(18)_** **=** **3.00**, ***p*** **=** **0.008, dz** **=** **0.71**	55.50 ± 10.29	*t*_(17)_ = 1.90, *p* = 0.074, dz = 0.47
Observing (M ± SD)	9.37 ± 2.48	11.32 ± 2.06	***t*_(18)_** **=** **3.33**, ***p*** **=** **0.004, dz** **=** **0.77**	10.78 ± 3.15	***t*_(17)_** **=** **2.16**, ***p*** **=** **0.046, dz** **=** **0.52**
Describing (M ± SD)	11.00 ± 2.62	11.47 ± 2.48	*t*_(18)_ = 0.96, *p* = 0.348	11.06 ± 2.44	*t*_(17)_ = 0.61, *p* = 0.552
Act w Awareness (M ± SD)	10.53 ± 3.01	10.89 ± 3.13	*t*_(18)_ = 0.92, *p* = 0.368	11.06 ± 2.94	*t*_(17)_ = 0.84, *p* = 0.415
Non-judging (M ± SD)	11.79 ± 2.32	12.84 ± 2.67	***t*_(18)_** **=** **2.73**, ***p*** **=** **0.014, dz** **=** **0.63**	12.22 ± 2.73	*t*_(17)_ = 1.04, *p* = 0.312
Non-reactivity (M ± SD)	10.26 ± 2.21	10.95 ± 2.97	*t*_(18)_ = 1.51, *p* = 0.148	10.39 ± 3.03	*t*_(17)_ = 0.39, *p* = 0.704
**ISI**					
Total score (M ± SD)	16.42 ± 3.95	8.37 ± 4.19	***t*_(18)_** **=** **−7.53**, ***p*** **<** **0.001, dz** **=** **1.73**	11.72 ± 4.95	***t*_(17)_** **=** **−4.57**, ***p*** **<** **0.001, dz** **=** **1.11**
Remission ( ≤ 7; *n*, %)	–	11/19; 57.9%	–	3/18; 16.7%	–
ISI ≥ 11 (*n*; %)	19/19; 100%	7/19; 36.8%	–	11/18; 61.1%	–
**Nocturnal wakefulness**					
SL (mins; M ± SD)	46.05 ± 34.24	16.32 ± 10.39	***t*_(18)_** ** = −3.64**, ***p*** **=** **0.002, dz** **=** **0.96**	32.22 ± 31.35	*t*_(17)_ = −1.53, *p* = 0.146
WASO (mins; M ± SD)	41.05 ± 28.41	28.95 ± 24.13	***t*_(18)_** **=** **−2.25**, ***p*** **=** **0.037, dz** **=** **0.52**	30.00 ± 30.77	***t*_(17)_** **=** **−2.35**, ***p*** **=** **0.031, dz** **=** **0.56**
TST (hrs; M ± SD)	5.45 ± 1.17	6.26 ± 0.84	***t*_(18)_** **=** **3.70**, ***p*** **=** **0.002, dz** **=** **0.88**	5.83 ± 1.20	*t*_(17)_ = 1.37, *p* = 0.189
**PHQ-9 (M** ±**SD)**					
Full sample (*n* = 19)	6.42 ± 3.47	3.32 ± 2.93	***t*_(18)_** **=** **−4.04**, ***p*** **=** **0.001, dz** **=** **0.93**	5.44 ± 4.44	*t*_(17)_ = −1.41, *p* = 0.178
Baseline PHQ ≥ 5 (*n* = 12)	8.33 ± 2.90	4.00 ± 3.46	***t*_(11)_** **=** **−4.17**, ***p*** **=** **0.002, dz** **=** **1.21**	6.75 ± 4.67	*t*_(11)_ = −1.34, *p* = 0.208
Baseline PHQ ≥ 10 (*n* = 4)	12.00 ± 0.82	5.50 ± 4.80	*t*_(3)_ = −3.09, *p* = 0.054, dz = 3.41	8.75 ± 6.40	*t*_(3)_ = −1.15, *p* = 0.335
PHQ-9 ≥ 10 (*n*, %)	4/19; 21.1%	1/19; 5.3%	–	4/18; 22.2%	–
PSASC (M ± SD)	22.79 ± 6.25	16.68 ± 5.72	***t*_(18)_** **=** **−5.57**, ***p***** < 0.001, dz** **=** **1.30**	19.17 ± 7.19	***t*_(17)_** **=** **−2.65**, ***p*** **=** **0.017, dz** **=** **0.63**
PSASC ≥ 19 (*n*;%)	13/19; 68.4%	4/19; 21.1%	**–**	10/18; 55.6%	**–**
PTQ (M ± SD)	42.89 ± 11.95	35.74 ± 9.98	***t*_(18)_** **=** **−4.76**, ***p*** **<** **0.001, dz** **=** **0.86**	36.83 ± 12.32	***t*_(17)_** **=** **−2.85**, ***p*** **=** **0.011, dz** **=** **0.68**
DISRS (M ± SD)	37.12 ± 10.13	31.25 ± 11.00	***t*_(18)_** **=** **−3.53**, ***p*** **=** **0.002, dz** **=** **0.82**	33.56 ± 12.65	***t*_(17)_** **=** **−2.60**, ***p*** **=** **0.019, dz** **=** **0.66**

t-statistic, p, and Cohen's dz correspond to the test statistic, significance value, and effect size associated with paired samples t-tests. Bold values indicate significant results. FFMQ-15 = 15-item version of the Five Facet Mindfulness Questionnaire. ISI, insomnia severity index. Remission is operationalized as ISI scores of ≤ 7 at posttreatment and follow-up. ISI ≥ 11 indicates clinically significant insomnia symptoms. SL, sleep latency; WASO, wake after sleep onset; TST, total sleep time; PHQ-9, patient health questionnaire-9. PHQ ≥ 5 indicates mild or worse depressive symptoms. PHQ ≥ 10 is a validated cutoff for detecting major depression. PSASC, pre-sleep arousal scale, cognitive factor. PSASC ≥ 19 indicates high nocturnal arousal. PTQ, perseverative thinking questionnaire; DISRS, daytime insomnia symptom response scale.

### MBTI effects on sleep

#### Acute effects: Pretreatment to posttreatment

Next, we examined MBTI effects on insomnia symptom severity and sleep parameters (Table 3). A paired samples *t*-test revealed a very large acute reduction in ISI in the full sample (Cohen's dz = 1.73). After treatment, 57.9% (*n* = 11/19) of patients remitted. Patient reports of sleep latency decreased by 30 mins, wake after sleep onset decreased by 12 mins, and total sleep time increased by 49 mins.

To examine whether Rx sleep aid use affects MBTI acute effectiveness on insomnia symptoms, we conducted a *post-hoc* paired samples *t*-test examining ISI changes within current Rx sleep aid users. Before treatment, ISI mean (±SD) was 15.50 ± 4.04, which decreased to 7.25 ± 4.03 at posttreatment [*t*_(3)_ = −6.27, *p* = 0.008, Cohen's dz = 1.86). This reduction resulted in *n* = 3/4 (75.0%) of these patients remitting.

#### Long-term effects: Pretreatment to follow-up

ISI scores at 6-month follow-up remained significantly lower than pretreatment levels. Notably, the effect size decreased in magnitude, but remained a very large effect (Cohen's dz = 1.11). Despite insomnia levels remaining lower than pretreatment, remission rates decreased to just 16.7% (*n* = 3/18). At follow-up, wake after sleep onset remained 11 mins shorter than pretreatment levels. However, no significant long-term effects were observed for sleep latency or total sleep time.

To examine whether Rx sleep aid use affects MBTI long-term effectiveness, we conducted a *post-hoc* paired samples *t*-test examining ISI changes in the patients with current Rx sleep aid use. Despite large acute reductions and a 75.0% remission rate at posttreatment, ISI scores at follow-up were unchanged from pretreatment levels [15.50 ± 4.04 to 15.00 ± 3.64; *t*_(3)_ = −0.32, *p* = 0.769] and 0.0% (*n* = 0/4) of these patients reported insomnia remission 6 months after treatment.

### *Post-hoc* analysis: Does race, obesity, or OSA-risk moderate MBTI effects on insomnia?

Post-hoc analyses showed that neither race, obesity, nor OSA-risk moderated treatment gains in MBTI. See Supplementary material for full results.

### *Post-hoc* descriptives: Rates of sleep aid use at posttreatment and follow-up

#### Rx sleep aids

Before treatment, four patients reported current Rx sleep aid use (Table 1). These four patients continued reporting Rx sleep aid use at both posttreatment and 6-month follow-up. No additional patients reported new Rx sleep aid use at these time-points.

#### OTC sleep aids

Before treatment, seven patients reported current OTC sleep aid use; five of these patients reported using ≥ 2 OTC sleep aids. After MBTI, the number of OTC sleep aid users decreased to three, and none of these patients reported taking more than one OTC sleep aid. Rates of OTC sleep aid use increased slightly at follow-up such that four patients reported OTC sleep aid use, and two of these patients reported using ≥ 2 OTC sleep aids.

### MBTI effects on depression

#### Acute effects: Pretreatment to posttreatment

We next conducted three paired samples *t*-tests to test acute effects of MBTI on PHQ-9 scores in the full sample, in patients with at least mild depression before treatment (PHQ-9 ≥ 5; *n* = 12/19), and in patients who screened positive for major depression before MBTI (PHQ-9 ≥ 10; *n* = 4/19). Results across all three tests consistently showed that MBTI produced large or very large reductions in depression (Table 3). Before treatment, 21.1% (*n* = 4/19) screened positive for major depression, but this rate decreased to 5.3% (*n* = 1/19) after treatment.

#### Long-term effects: Pretreatment to follow-up

Paired samples *t*-tests did not reveal significant long-term effects of MBTI on depression (Table 3). Notably, 6 months after MBTI, 22.2% (*n* = 4/18) of patients screened positive for major depression, which was similar to pretreatment rates.

### MBTI effects on cognitive arousal

#### Acute effects: Pretreatment to posttreatment

Paired samples *t*-tests revealed large to very large reductions in PSASC, PTQ, and DISRS at posttreatment (Table 3). Descriptive data showed that 68.4% (*n* = 13/19) of patients reported high nocturnal cognitive arousal before MBTI, whereas just 21.1% (*n* = 4/19) reported high cognitive arousal at posttreatment.

#### Long-term effects: Pretreatment to follow-up

Paired samples *t*-tests showed that reductions in PSASC, PTQ, and DISRS remained statistically significant at follow-up, but that effect sizes decreased to medium-to-large effects (Table 3). Descriptive data showed that high nocturnal cognitive arousal rates increased from 21.1% (*n* = 4/19) at posttreatment to 55.6% (*n* = 10/18) at 6-month follow-up.

### Post-hoc: Identifying potential treatment mechanisms of MBTI for insomnia and depression

Lastly, we explored whether pretreatment to posttreatment changes in insomnia and depression were associated with changes in cognitive arousal and mindfulness (Table 4). Regarding insomnia outcomes, we observed that reductions in ISI were strongly correlated with reductions in PSASC, and the association between changes in PTQ and ISI approached significance (*p* = 0.063). Regarding depression, we observed that reductions in PHQ were significantly correlated with reductions in PSASC and DISRS, whereas associations with PTQ (*p* = 0.095) and FFMQ (*p* = 0.078) approached significance. Notably, changes in ISI and PHQ were also significantly correlated.

**Table 4:** Bivariate correlations between pretreatment to posttreatment changes in insomnia, depression, cognitive arousal indices, and everyday mindfulness.

	**Δ ISI**		**Δ PHQ**
Δ PSASC	0.709^***^	Δ PSASC	0.528^*^
Δ PTQ	0.435^a^	Δ PTQ	0.394^a^
Δ DISRS	0.205	Δ DISRS	0.519^*^
Δ FFMQ	−0.044	Δ FFMQ	−0.414^a^
Δ PHQ	0.562^*^	Δ ISI	0.562^*^

*** p < 0.001;

* p < 0.05.

a = 0.05 < p < 0.10.

## Discussion

The present study was a single-arm proof-of-concept clinical trial examining the acute and long-term effects of telemedicine MBTI as a second-stage treatment on symptoms of insomnia, depression, and cognitive arousal in 19 patients who did not adequately respond previously to CBTI and/or pharmacotherapy. Patients were highly engaged in and adherent to MBTI when delivered individually *via* telemedicine, which produced moderate and durable increases in everyday mindfulness. Moreover, MBTI produced large acute reductions in insomnia, depression, and cognitive arousal in these patients who did not adequately respond to prior CBTI and/or pharmacotherapy. Evidence also supported the durability of MBTI effects on insomnia and cognitive arousal in this treatment-resistant population, although effect sizes decreased over the long-term. Unfortunately, antidepressant effects were not observed 6 months after treatment. Taken together, MBTI may represent a viable option for patients with a history of non-remission in response to CBTI and/or pharmacotherapy, although patients with a history of treatment-resistance may benefit from longer-term care to help maintain initial gains.

### MBTI improves sleep, but treatment gains diminish over time in treatment-resistant insomnia

The acute effects of MBTI on insomnia symptoms, sleep latency, wake after sleep onset, and total sleep time were very large. Moreover, mean values for each of these metrics were within normal limits after treatment. Indeed, 57.9% of patients achieved insomnia remission at posttreatment, which is especially notable since *n* = 16/19 of patients received two previous doses of CBTI without achieving remission. These very large acute effects on insomnia observed in this single-arm trial are consistent with prior evidence from RCTs showing MBTI to produce very large reductions in insomnia symptoms (Ong et al., 2014; Perini et al., 2021). Importantly, preliminary evidence suggests that MBTI may be effective regardless of race, obesity, or OSA-risk. However, given our small sample size, future research utilizing larger samples is needed to determine whether these findings replicate.

Most patients in this study used some form of Rx and/or OTC sleep aid before treatment. Importantly, MBTI was acutely effective among patients with long-term Rx sleep aid use. Specifically, 75.0% of long-term Rx sleep aid users remitted from insomnia after MBTI. Even so, we saw no change in Rx sleep aid use in this sub-sample despite improvements in sleep. Along these lines, the number of OTC sleep aid users decreased from seven before treatment to three patients after MBTI. Future studies using an RCT design should examine whether MBTI can reduce reliance on sleep aid medications for insomnia patients with poor response to pharmacotherapy.

Despite these initial gains, some treatment-resistant patients experienced recurrence of insomnia symptoms in the 6 months following MBTI. This resulted in a remission rate of just 16.7% at 6-month follow-up, despite insomnia symptom levels at follow-up remaining significantly lower than pretreatment levels. Thus, although our study findings support durability of MBTI effects, we observed that the magnitude of long-term effects insomnia may be lessened in treatment-resistant patients, especially those with long-term Rx sleep aid use. Despite an initial 75.0% remission rate at the end of treatment, all Rx sleep aid users relapsed within 6 months after completing treatment.

It is important to highlight that the decrease in MBTI effects on insomnia symptoms across long-term follow-up is unique to our sample of patients with a history of poor response to insomnia psychotherapy and pharmacotherapy. By comparison, a previous RCT in a treatment-naïve sample of insomnia patients showed that the magnitude of MBTI effects on insomnia actually increased in the 6 months following treatment (Posttreatment Cohen's d = 2.07; 6-month follow-up Cohen's d = 2.56); 50.0% of patients were in remission 6 months after completing MBTI (Ong et al., 2014). Taken together, these data suggest that MBTI has durable effects on insomnia, but that durability may be reduced in patients with treatment-resistant insomnia.

### MBTI produces short-term antidepressant effect in treatment-resistant patients

Patients in our trial reported very large reductions in depressive symptoms, particularly among those who screened positive for major depression before treatment. Notably, depression rates decreased from 21.1% at baseline to 5.3% after MBTI. Acute antidepressant effects were larger in this study relative to a previous RCT wherein MBTI produced small-to-moderate effects on depression (Ong et al., 2018), whereas antidepressant effects have been observed to be medium to large in other sleep-disturbed patient samples when treated with Mindful Awareness Practices (MAPs) (Black et al., 2015) or MBSR (Zhang et al., 2015).

Despite large acute reductions in depression, the antidepressant effects were not long-lasting. Six months after treatment, depression symptoms returned to pretreatment levels, once again affecting 1 in 5 patients. Although no prior clinical trials have tested the long-term effects of MBTI on comorbid depression, it is worth noting that acute antidepressant effects in mindfulness-based interventions are robust, but that longer-term effects on depression have limited empirical support, partly due to few studies examining long-term antidepressant effects of mindfulness-based interventions (Chi et al., 2018; Goldberg et al., 2019; Li and Bressington, 2019).

### MBTI produces durable reductions in cognitive arousal

We have previously identified that reducing cognitive arousal is a key mechanism by which insomnia therapy can alleviate insomnia and depression, but also that RCTs examining traditional CBTI have produced mixed effects on cognitive arousal including small or even non-significant effects (Vincent and Walsh, 2013; Espie et al., 2014; Kalmbach et al., 2019a, 2022c; Cheng et al., 2020). In the present study, we observed very large acute reductions in nocturnal cognitive arousal. Indeed, nearly 70% of patients reported high nocturnal cognitive arousal before MBTI, but this rate decreased to just 21% after treatment. Notably, MBTI had broad effects on cognitive arousal including transdiagnostic pre-sleep cognitive activity and general perseverative thinking, as well as disorder-specific insomnia-focused rumination.

Importantly, these findings add further support to the conceptual basis of mindfulness-based interventions, such as MBTI, as targeting cognitive arousal in the context of insomnia. The large acute reductions observed in our trial replicate findings from prior clinical trials showing that MBTI produces large reductions in nocturnal cognitive arousal and cognitive sleep effort (Ong et al., 2008, 2014, 2018; Perini et al., 2021). Moreover, our patients with low cognitive arousal reported higher rates of insomnia remission and lower rates of depression relative to patients with high cognitive arousal at posttreatment (insomnia remission: 67% vs. 25%; depression: 0% vs. 25%) and 6-month follow-up (insomnia remission: 38% vs. 0%; depression: 0% vs. 40%).

Additionally, the large acute effects of MBTI on cognitive arousal add to the field by demonstrating that treatment-resistant insomnia patients respond favorably to MBTI for reducing cognitive arousal. As persistent and refractory arousal has been linked to poor CBTI and Rx sleep aid response in multiple prior studies (Pillai et al., 2016; Kalmbach et al., 2019a, 2022c; Cheng et al., 2020), MBTI and other mindfulness-based interventions may serve as potentially viable alternative treatment options for insomnia patients, especially for those who have not adequately responded to CBTI and/or pharmacotherapy.

MBTI also produced significant long-term reductions in all three cognitive arousal indices 6 months after treatment, further supporting the therapeutic benefits of MBTI for reducing cognitive arousal in treatment-resistant insomnia. Important to emphasize, however, is that the magnitude of treatment gains declined following treatment despite remaining significantly lower than pretreatment levels. That is, 6 months after MBTI, approximately half of patients reported high cognitive arousal (up from 21% at posttreatment). This reduction in effect size is in contrast to a prior MBTI RCT showing that reductions in arousal remained large at 3 and 6 months after treatment in a sample of treatment-naïve patients (Ong et al., 2014).

### How should treatment-resistant insomnia be addressed?

CBTI is a highly effective first-line treatment for insomnia, but not all patients adequately respond (Trauer et al., 2015; Wu et al., 2015; Qaseem et al., 2016; van der Zweerde et al., 2019). Additionally, pharmacotherapy is a highly common treatment for insomnia—especially in primary care, where insomnia is often treated—despite weaker empirical support for its effectiveness (Qaseem et al., 2016; Grandner and Chakravorty, 2017). Recently, Morin et al. (2020) showed that sequential treatment involving psychological and pharmacological therapies is an effective strategy for managing insomnia. Specifically, they found that insomnia response rates significantly improved when behavioral therapy was followed by cognitive therapy or zolpidem for initial non-remitters.

### MBTI may represent a viable second-stage therapy

Results from the present study add to the literature by showing that insomnia patients who do not remit with CBTI and/or pharmacotherapy may benefit from MBTI. It is important to highlight that, in the previous sequential treatment RCT, neither behavior therapy nor trazodone significantly improved treatment outcomes for patients who first failed to respond to zolpidem (Morin et al., 2020). However, our study offers preliminary evidence to suggest that MBTI may be effective in acutely helping Rx sleep aid users with poorly managed insomnia. These findings offer patients and providers a treatment option to improve insomnia management in those who do not adequately respond to pharmacotherapy. Notably, population data suggests that Rx sleep aid use is largely driven by hyperarousal (Pillai et al., 2016). Thus, it is possible that Rx sleep aid users benefit from the large MBTI effect on cognitive arousal observed in our study.

### Identifying key treatment mechanisms

To best address treatment-resistant insomnia, we must identify potential mechanisms facilitating reductions in insomnia and comorbid depression. Prior research shows mindfulness-based interventions effectively reduce cognitive arousal, particularly in the forms of perseverative thinking (Deyo et al., 2009; Campbell et al., 2012; Querstret and Cropley, 2013; Creswell, 2017; Renna et al., 2017, 2018; Winnebeck et al., 2017). Moreover, focus group data show that poor sleepers show interest in mindfulness-based programs to help alleviate worry (Felder et al., 2022). These prior findings align with *post-hoc* exploration of the present study's data showing that reductions in insomnia and depression were strongly associated with reductions in cognitive arousal, particularly nocturnal cognitive arousal. Given that cognitive arousal increases both insomnia and depression (Kalmbach et al., 2021), we believe these data indicate that reducing cognitive arousal—especially at night—enhances insomnia and depression outcomes.

To illustrate: a patient presents to therapy with insomnia and depression. When exposed to stress, the patient responds by worrying and ruminating, which increases insomnia and depression symptoms. However, after participating in a mindfulness program, they learn to respond to stress mindfully and more adaptively rather than ruminating or worrying uncontrollably, thereby reducing insomnia and depression symptoms. Indeed, this interpretation aligns with recent prospective observational data showing that rumination mediates effects of mindfulness on depression symptoms such that higher levels of mindfulness predicted lower rumination, which, in turn, predicted lower depression (Jury and Jose, 2019). Future randomized controlled trials should consider evaluating reductions in cognitive arousal and increases in mindfulness as potential treatment mechanisms for alleviating insomnia and depression.

### Limited long-term gains for treatment-resistant patients

Despite strong acute effects, treatment gains decreased within 6 months of completing MBTI in this treatment-resistant population, especially for those using Rx sleep aids. Thus, consideration should be given to discovering how to best maintain initial treatment gains. These options may include elements to promote continued meditation practice and/or implementation of behavioral sleep strategies. Unfortunately, long-term follow-up and relapse prevention has been understudied in both insomnia and depression research. Even so, relapse prevention strategies that include creating a relapse prevention plan with patients, proactive monitoring and follow-up (including regular use of tools for tracking deterioration), and/or provision of planned booster sessions after acute treatment (Moriarty et al., 2020) should be considered for optimizing long-term outcomes in treatment-resistant insomnia patients.

Notably, CBTI and MBTI include creating a relapse prevention plan with patients at the end of treatment. These plans include education on recognizing early signs of relapse and how to re-implement strategies for addressing these early symptoms. However, this approach requires the patient to take an active role in relapse prevention (noticing early symptoms and implementing the correct strategies), whereas providers take a completely passive role. Unfortunately, this relapse prevention strategy is insufficient for some patients, and treatment-resistant patients may especially benefit from relapse prevention strategies that utilize greater provider support. Future studies would benefit from testing relapse preventions strategies involving proactive monitoring and/or planned booster sessions vs. treatment as usual.

*Long-term care: Clinician-led options* for long-term maintenance may include continued individual care with a behavioral sleep medicine clinician on a reduced frequency (e.g., monthly). Additionally, mindfulness retreats for MBTI alumni may also help promote continued long-term practice. Importantly, due to present real-world logistical limitations with regard to MBTI accessibility, alternate options for long-term care may include group-based MBSR. MBSR is widely available, effective for broad patient populations, and promotes continued mindfulness practice and improved sleep (Ong et al., 2014, 2018). Indeed, MBSR improves wellbeing and reduces stress in healthy individuals and across a wide range of mental health and physical health conditions (Grossman et al., 2004; Khoury et al., 2015). Therefore, MBSR may present a viable option for longer-term insomnia maintenance after initial remission with MBTI. It is also possible that sequencing MBTI with motivational interviewing or motivational enhancement therapy may promote long-term insomnia maintenance by evoking patients' intrinsic reasons and desires for continued and independent meditation practice (Garland and Howard, 2018).

*Long-term care: Digital health therapeutics (also known as eHealth)* may also play a role in maintaining gains over the long-term. Digital care may involve using CBTI-based digital intervention to continue monitoring of sleep symptoms and recommendations of behavioral sleep strategies. However, currently available CBTI programs are largely designed for acute treatment rather than long-term maintenance. Alternatively, mindfulness-based digital programs—including guided meditation apps and MBSR-based apps—could potentially play a role in promoting continued mindfulness practice and, by extension, keep cognitive arousal levels low. Indeed, high cognitive arousal after MBTI increases risk for insomnia relapse (Ong et al., 2009), therefore long-term insomnia management programs would benefit from targeting cognitive arousal even after acute alleviation.

### Limitations and future directions

The present proof-of-concept study should be interpreted considering certain limitations. Our primary limitation concerns our reliance on patient-reported adherence metrics. Future trials leveraging a mindfulness meditation app that allows researchers to examine objective data regarding frequency and duration of meditations would provide more accurate adherence metrics. Along these lines, larger-scale studies should examine whether objective and/or subjective adherence metrics predict treatment outcomes. Because of the small sample in this study and restricted range due to high reported compliance, the present study was underpowered to determine whether adherence predicted study outcomes.

Moreover, a second—and related—limitation is that we did not assess patient mindfulness practices or sleep-wake schedules between posttreatment and follow-up. Therefore, we were unable to test whether reductions in meditation time and/or straying from the prescribed sleep-wake period were associated with worsening symptoms over the longer-term. Additionally, as this study was a single-arm trial, we are unable to determine the extent to which treatment effects are attributable to the introduction of mindfulness strategies vs. the consolidation of behavioral sleep strategies learned previously by some patients that are also included in MBTI. A limitation related to study outcomes is the observed poor internal consistency for the FFMQ-15's observing scale, which may have resulted in underestimation of MBTI effects on observing outcomes.

Although we outlined our conceptual basis for testing MBTI as second-stage therapy, we also wish to acknowledge that other interventions may serve as viable options. Specifically, cognitive therapy for insomnia (Harvey, 2005), MBSR (Kabat-Zinn, 1982), MAPs (Zylowska, 2012), and other Rx sleep aids are among other potential second-stage treatment options that may reduce insomnia, depressive symptoms, and cognitive arousal in this treatment-resistant patient population. Finally, we must highlight that our sample only consisted of patients who identified as non-Hispanic Black and non-Hispanic White, which limits the generalizability of our findings.

As this was a proof-of-concept study, we believe our findings strongly support the scientific premise for testing the efficacy of MBTI as a second-stage therapy in a RCT including a control condition and/or alternative active comparator. Moreover, our findings support future investigations to explore and identify feasible and effective longer-term care strategies to help maintain acute treatment gains in this treatment-resistant patient population.

### Conclusions

Our study supports the concept and feasibility of MBTI for patients who previously failed CBTI and/or pharmacotherapy. Patients were highly engaged in and adherent to MBTI. We observed large acute effects on insomnia, depression, and cognitive arousal regardless of race, obesity, or OSA-risk. Although MBTI is traditionally delivered *via* in-person group format, trial results support its effectiveness when delivered in an individual format using telemedicine. Moreover, we demonstrated that MBTI can be effectively delivered by nurses, which supports its scalability using non-mental health professionals. Despite large acute reductions in clinical symptoms, the magnitude of effects decreased within 6 months after treatment. By extension, treatment-resistant patients may benefit from continued care to help maintain initial treatment gains and to reduce the risk for insomnia and depression relapse.

## Data availability statement

The raw data supporting the conclusions of this article will be made available by the authors, without undue reservation.

## Ethics statement

The studies involving human participants were reviewed and approved by Henry Ford Health Institutional Review Board. The patients/participants provided their written informed consent to participate in this study.

## Author contributions

CD was the principal investigator for the study. CD and DK contributed to the study conceptualization and analysis. DK conducted data analysis and drafted the manuscript. CD, DK, and PC contributed to data interpretation and reporting. CF-C, MR, CS, and ZS contributed to study protocol facilitation and study completion. CD, PC, JO, AR, DF, and MR contributed to editing and proofreading the manuscript. All authors approved the submitted version.
