# Supplementary material for: Mindfulness-based therapy for insomnia alleviates insomnia, depression, and cognitive arousal in treatment-resistant insomnia: A single-arm telemedicine trial
Source: Front Sleep. 2023 Mar 8;2:1072752. doi: 10.3389/frsle.2023.1072752 (PMC12713880; doi:10.3389/frsle.2023.1072752)
Supplement: Supplementary file 1 [file Presentation_1.pdf]

Supplementary Materials

Posthoc analysis: Does race, obesity, or OSA-risk moderate MBTI effects?

Race. Seven patients identified racially as non-Hispanic Black, whereas nine patients identified as non-Hispanic White. Patients who self-identified as Black reported a reduction of 9.00±5.29 points on the ISI, whereas patients who self-identified as White reported a reduction of 7.50±4.40 points. An independent samples t-test showed that these change scores did not significantly differ between groups, $t(17)=0.67$, $p=.514$.

Obesity. Five patients had BMI $\geq$ 30, whereas 14 had BMI < 30. Patients with BMI $\geq$ 30 reported a reduction of 10.20±3.70 points on the ISI, whereas patients at low risk for OSA reported a reduction of 7.29±4.84 points. An independent samples t-test showed that these change scores did not significantly differ between groups, $t(17)=1.22$, $p=.241$.

OSA-risk. Nine patients screened positive for high OSA risk on the STOP-BANG ($\geq$ 3), whereas 12 patients did not. Patients at high risk for OSA reported a reduction of 9.67±5.61 points on the ISI, whereas patients at low risk for OSA reported a reduction of 6.60±3.24 points. An independent samples t-test showed that these change scores did not significantly differ between groups, $t(17)=1.48$, $p=.158$.
